# Lifesaving emergency obstetric services are inadequate in south-west Ethiopia: a formidable challenge to reducing maternal mortality in Ethiopia

**DOI:** 10.1186/1472-6963-13-459

**Published:** 2013-11-04

**Authors:** Meseret Girma, Yaliso Yaya, Ewenat Gebrehanna, Yemane Berhane, Bernt Lindtjørn

**Affiliations:** 1 Department of Public Health, College of Medicine and Health Sciences, Arba Minch University, Arba Minch, Ethiopia; 2 Centre for International Health, University of Bergen, Bergen, Norway; 3 Department of Reproductive Health and Nutrition, Addis Continental Institute of Public Health, Addis Ababa, Ethiopia; 4 Arba Minch College of Health Sciences, Arba Minch, Ethiopia

## Abstract

**Background:**

Most maternal deaths take place during labour and within a few weeks after delivery. The availability and utilization of emergency obstetric care facilities is a key factor in reducing maternal mortality; however, there is limited evidence about how these institutions perform and how many people use emergency obstetric care facilities in rural Ethiopia. We aimed to assess the availability, quality, and utilization of emergency obstetric care services in the Gamo Gofa Zone of south-west Ethiopia.

**Methods:**

We conducted a retrospective review of three hospitals and 63 health centres in Gamo Gofa. Using a retrospective review, we recorded obstetric services, documents, cards, and registration books of mothers treated and served in the Gamo Gofa Zone health facilities between July 2009 and June 2010.

**Results:**

There were three basic and two comprehensive emergency obstetric care qualifying facilities for the 1,740,885 people living in Gamo Gofa. The proportion of births attended by skilled attendants in the health facilities was 6.6% of expected births, though the variation was large. Districts with a higher proportion of midwives per capita, hospitals and health centres capable of doing emergency caesarean sections had higher institutional delivery rates. There were 521 caesarean sections (0.8% of 64,413 expected deliveries and 12.3% of 4,231 facility deliveries). We recorded 79 (1.9%) maternal deaths out of 4,231 deliveries and pregnancy-related admissions at institutions, most often because of post-partum haemorrhage (42%), obstructed labour (15%) and puerperal sepsis (15%). Remote districts far from the capital of the Zone had a lower proportion of institutional deliveries (<2% of expected births compared to an overall average of 6.6%). Moreover, some remotely located institutions had very high maternal deaths (>4% of deliveries, much higher than the average 1.9%).

**Conclusion:**

Based on a population of 1.7 million people, there should be 14 basic and four comprehensive emergency obstetric care (EmOC) facilities in the Zone. Our study found that only three basic and two comprehensive EmOC service qualifying facilities serve this large population which is below the UN’s minimum recommendation. The utilization of the existing facilities for delivery was also low, which is clearly inadequate to reduce maternal deaths to the MDG target.

## Background

The fifth Millennium Development Goal (MDG 5) is to reduce maternal mortality by 75% between 1990 and 2015. Although there are good tools available to help reduce maternal deaths [1], the limited availability and poor quality of services cause nearly 300,000 maternal deaths in the world every year, with approximately 85% of the 287,000 global maternal deaths taking place in both Sub-Saharan Africa (56%) and southern Asia (29%) [2]. In 2008, more than half of all maternal deaths in the world occurred in six countries: Afghanistan, Democratic Republic of the Congo, Ethiopia, India, Nigeria and Pakistan [3], with most of these preventable and unacceptable deaths occurring around delivery or a few days after [4]. Bleeding during pregnancy and birth, obstructed and prolonged labour and pregnancy-related hypertension represent the leading causes of deaths among women of reproductive age in resource-poor countries [5].

The maternal mortality ratio (MMR) for Ethiopia was 1,061 (665–1,639) in 1980, 968 (600–1,507) in 1990, 937 (554–1,537) in 2000 and 590 (358–932) in 2008 [3]. Nevertheless, the results of the 2011 Demographic and Health Survey (DHS) revealed that there has been little progress in reducing maternal mortality [6]. The DHS estimate of the MMR for 2011was 676 (541–810) per 100,000 live births. A study has also showed that in sub-Saharan African countries, the progress towards achieving MDG5 has been slow because of a poor quality of care, low access, inadequate skilled personnel and financial barriers to care [3].

The WHO recommends the use of process indicators on emergency obstetric care (EmOC) facilities to assist in monitoring the progress in maternal mortality reduction efforts, which are considered necessary for planning, implementing and monitoring initiatives to improve maternal health [7]. Unfortunately, there is limited evidence regarding how these institutions are distributed, how well the existing facilities perform and how many people use them in Gamo Gofa, Ethiopia. The investment in maternal health programmes can be evaluated by measuring input indicators (midwifery training), process (the number of midwives posted) and outcomes (the uptake of skilled delivery care). However, the assessment of impacts such as the reduction in mortality in a community can show the effects of long-term interventions.

The availability and use of emergency obstetric care services is important for reducing maternal morbidity and mortality. Based on the capacity to provide lifesaving emergency obstetric procedures, a health institution can be classified as basic or comprehensive emergency obstetric care facility [8]. Basic EmOC institutions are expected to provide the following six services (signal functions): administration of parenteral antibiotics, parenteral oxytocic drugs, parenteral anticonvulsants for pre-eclampsia, manual removal of retained placentas, removal of retained products of conception and assisted vaginal delivery (vacuum extractions or forceps deliveries) [8]. Institutions providing comprehensive EmOC should also be capable of performing caesarean sections, blood transfusions and services provided by the basic EmOC institutions.

The findings of an assessment regarding the availability, quality and distribution of EmOC services is important for health professionals and policymakers involved in maternal health services. With high maternal mortality rates and a mostly rural population, it is important to evaluate emergency obstetric care provided at public health institutions in Ethiopia.

A recent study has shown there are too few health institutions providing EmOC to meet the UN standards of at least five (four basic and one comprehensive) EmOC institutions per 500,000 population in Ethiopia [9]. Only 7% of deliveries took place in institutions, including only 3% in institutions that routinely provided all signal functions. Six percent of women with obstetric complications were treated in health institutions, whereas only one-half of these women were treated in fully functional comprehensive EmOC facilities [9]. The study concluded that far too few public institutions in Ethiopia meet the indicators set by the UN standards.

Ethiopia therefore faces many challenges, not only because of a limited number of adequately functioning obstetric facilities, but also because of its large population and mountainous topography, with large parts of the populations living in remote areas. We conducted this study to assess the availability (coverage), quality (functionality) and utilization of emergency obstetric care facilities in Gamo Gofa in south-west Ethiopia.

## Methods

### Setting

**Figure 1:**
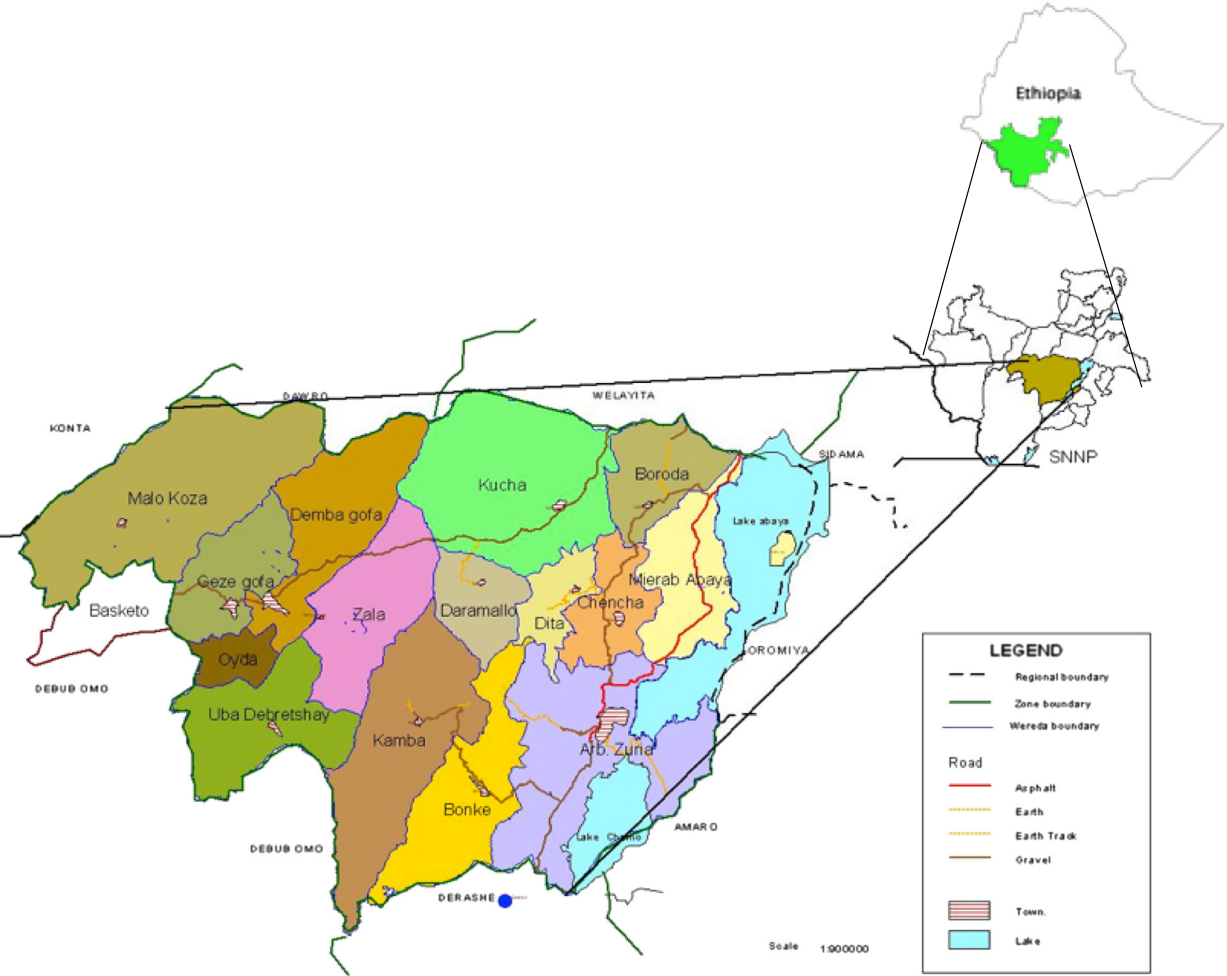
Administrative map of Gamo Gofa Zone and its Woredas, south-western Ethiopia, 2010.

The study was conducted in the Gamo Gofa Zone in south-west Ethiopia (see map in Figure 1). Nearly 1.7 million people live in the area, with 90% living in rural communities. The Zone has 15 woredas (districts) and two town administrations, each being directly administratively responsible to the Zone. However, people in the surrounding districts of the towns, as well as the towns themselves, use the health facilities/services/ in these towns. The Zone represents three climatic zones (cold, temperate and hot), where most of the people live in highlands 2,000 metres above sea level and practice subsistence farming. There are few all-weather roads in the area, although most of the population lives in the highlands without access to roads. Health care is provided by three hospitals, 63 health centres and by rural health extension workers in 483 kebeles, which are Ethiopia’s lowest administrative units, with an average coverage of 1,000 households (population of 5,000). Hospitals are expected to provide comprehensive emergency obstetric care, while the health centres are expected to provide basic emergency obstetric care. Due to limited access to hospitals, senior staff (health officers) are given minimal training, and provide services such as caesarean sections in some health centres. Four (6%) of the health institutions in the area are accessible by asphalt roads, 21 facilities (32%) are accessed by all-weather gravel roads, 30 health centres (46%) are only accessible by car during the dry season and 11 institutions (17%) could not be accessed by a vehicle at the time of the survey.

We conducted this study as part of a public health intervention project aimed at reducing maternal mortality in Gamo Gofa. A few years prior to the study, the intervention programme (“Reducing Maternal Mortality in south-west Ethiopia”) had started training non-physician clinicians (NPCs) to provide EmOC, including caesarean sections. The programme aims to support public health services to help reduce maternal and neonatal deaths [10], and is primarily a support to government institutions with training, supervision and providing the institutions with basic equipment. Thus, while the population in 2007 had only one hospital capable of doing comprehensive EmOC for approximately 1.7 million people, the services such as caesarean section delivery had improved to three hospitals and two health centres (one institution per 350,000 people) by 2010. The project also includes studies on estimating maternal and neonatal mortality through community-based birth registries, estimations of maternal mortality through the sisterhood method, large-sample household survey to estimate maternal and neonatal deaths and a health facilities obstetric care quality study (the current study).

### Data collection and instruments

We collected data using questionnaires and procedures developed according to UN guidelines [8], and assessed the performance of health institutions using the same guidelines. We recruited eight health officers (people with bachelor’s degrees in clinical and community medicine) to collect the data, and the health officers were trained for two days before visiting the institutions. If deemed necessary, key health personnel at each institution were interviewed for the clarification of any recorded data.

Between September and November 2010, we visited 66 health institutions, the three hospitals in Arba Minch, Chencha and Sawla and 63 health centres throughout the Zone. When visiting the institutions, we retrospectively reviewed one year of available obstetric services, records, documents, cards and registration books related to delivery services. As a result, we collected information from records and registers such as admission registers, delivery registers, delivery log books, referral registers and death registers. We also registered the number of staff available for obstetric care at each of the health institutions we reviewed. As recommended by the WHO guidelines for areas with fewer than 100 facilities, we included all hospitals and health centres in Gamo Gofa in the current study [8].

### Data analysis

We used SPSS (version 16; SPSS, Inc., Chicago, IL, USA) for data entry and statistical analysis, and we performed a descriptive analysis to present rates and ratios. We calculated the expected number of deliveries for each woreda using the Central Statistical Authority (CSA) estimates for birth rates (3.7%) and woreda population size [11].

### Operational definition

An EmOC facility refers to whether or not an institution is fully functioning as a basic or comprehensive facility [8]. Functioning is defined by nine signal functions, as follows: administering parenteral antibiotics, administering parenteral oxytocic drugs, administering parenteral sedatives, manual removal of the placenta, removal of retained products of conception, vacuum-assisted vaginal deliveries or forceps deliveries, performing caesarean sections, performing newborn resuscitation and the availability of a blood transfusion service. An institution that had not performed any or only some of the signal functions during the past three months was defined as a non-functioning EmOC. The reasons for not performing signal functions may vary, and include a lack of equipment or medications or a lack of available skilled personnel.

### Ethical issues

The data for this study was collected as a part of Meseret Girma’s master thesis at the University of Gondar, so ethical clearance was therefore obtained from the University of Gondar. After obtaining the clearance, we received written permission to carry out the study from the Gamo Gofa Zone Health Department and each of the woreda health authorities. Before starting to record information about the health institutions, we informed the leaders of each of the health institutions about the study. Lastly, we received a written consent from the head of each facility to allow us to conduct the study at the institution. The Regional Committee for Medical and Health Research Ethics of North Norway (REK Nord) also approved this study.

## Results

### Availability of EmOC

**Figure 2:**
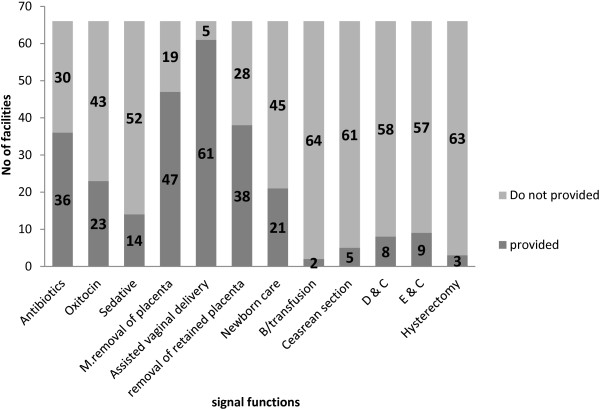
Signal functions provided at all health centres and hospitals in Gamo Gofa Zone between July 2009 and June 2010.

We visited and reviewed all of the 66 health institutions (hospitals and health centres) in Gamo Gofa. Of these, only the two hospitals in Arba Minch and Sawla (3% of institutions) provided all signal functions, and were thus designated as providing comprehensive EmOC. Three health centres (4.5%) provided basic EmOC, but did not have a blood bank, while 61 (92%) facilities lacked some or all signal functions and 40 (60.6%) institutions lacked > 5 of the signal functions. Only 36 (54.5%) institutions provided parenteral antibiotics when needed, 61 of 66 (92%) performed assisted vaginal deliveries, 47 (71%) performed the manual removal of placentas, 23 (35%) used parenteral oxytocin and 14 (21%) used anticonvulsants during eclampsia when indicated in the last three months (Figure 2).

### Delivery, complications, and deaths

A total of 4,231 deliveries and related admissions took place at the health institutions over the course of 1 year. Furthermore, there was an annual average of 522 deliveries at each hospital, 213 deliveries at two health centres capable of providing emergency obstetric care, including caesarean sections, and an average of 32 deliveries at each of the remaining 61 health centres. Five health centres did not have any recorded deliveries and 24 health centres had one delivery per month during the year surveyed. A total of 521 deliveries were done by caesarean section (0.8% of 64,413 expected births and 12.3% of 4,231 facility births), and over the one year, we recorded 10 neonatal deaths and 178 stillbirths.

We reviewed 1,031 of 4,231 (24.3%) births and pregnancy-related admissions at the health facilities as complicated cases. The complications were further categorized as complications associated with abortions (28.2%), obstructed labour (18%), prolonged labour (16.9%), post-partum haemorrhage (7.3%), antepartum haemorrhage (6.3%), pre-eclampsia or eclampsia (4%) and unclassified (7.5%). We recorded 79 maternal deaths, with the primary causes of deaths being haemorrhage (42%), obstructed labour (15%), puerperal sepsis (15%), prolonged labour (8%) and complications from abortions (8%, Table 1). Table 2 shows that the proportion of institutional deaths varied between districts. Very high mortality rates (61% and 28%) were recorded in two rural and remote woredas; these woredas also had very low institutional delivery rates, and few midwives worked at the institutions (Table 2).

**Table 1:** Major causes of pregnancy and birth complications and maternal deaths in hospitals and health centres in Gamo Gofa, south-west Ethiopia, July 2009 to June 2010

**Causes**	**Complications**		**Deaths**	
	**No.**	**%**	**No.**	**%**
Ante partum haemorrhage	65	6.3	1	1
Post-partum haemorrhage	75	7.3	33	42
Prolonged labour	174	16.9	6	8
Obstructed labour	186	18.0	12	15
Puerperal sepsis	57	5.5	10	13
Complication of abortion	291	28.2	6	8
Pre-eclampsia	41	4.0	4	5
Ruptured uterus	65	6.3	5	6
Others	77	7.5	2	3
Total	1,031	100	79	100

**Table 2:** Expected births, institutional deliveries and health human resource distributions for 66 health institutions, 2010, Gamo Gofa, south-west Ethiopia

**Woreda**	**Population**	**No. of institutions**	**Expected no. of births**^**§**^	**Institutional deliveries**		**Midwives**		**Nurses and health officers**^**†**^		**Doctors**^**†**^	**Maternal deaths**
				**No.**	**%**	**No.**	**Per 100,000 population**	**No.**	**Per 100,000 population**		**No.**
Melokoza	131,009	5	4,847	102	2.1	2	1.5	20	15.3	0	0
Denba Goffa**	114,309	5	4,230	417	9.9	11	9.6	8	7	1	17
Kucha	162,513	6	6,013	108	1.8	2	1.2	13	8	0	5
Boreda	74,008	4	2,738	30	1.1	5	6.8	22	29.7	0	1
Merab Abaya	81,819	4	3,027	287	9.5	4	4.9	22	26.9	0	5
Arba Minch Zuria*	264,927	7	9,802	1,809	18.5	17	6.4	54	20.4	5	3
Chencha	122,193	5	4,521	409	9	8	6.5	45	36.8	2	6
Dita	91,433	4	3,383	163	4.8	3	3.3	16	17.5	0	7
Daramalo	88,232	2	3,265	54	1.7	3	3.4	3	3.4	0	0
Zala	80,931	5	2,995	58	1.9	3	3.7	36	44.5	0	2
Ubadebretsehay	75,377	3	2,789	37	1.3	2	2.7	15	19.9	0	0
Kemba	169,756	7	6,281	411	6.5	4	2.4	13	7.7	0	3
Bonke	173,240	5	6,410	276	4.3	4	2.3	11	6.3	0	3
Geze Goffa	74,951	3	2,773	56	2	3	4	2	2.7	0	21
Oyida	36,187	1	1,339	14	1	2	5.5	1	2.8	0	6
Gamo Gofa Zone	1,740,885	66	64,413	4,231	6.6	73	4.2	281	16.1	8	79

*Includes Arba Minch Town; **Includes Sawla Town; ^†^Available for obstetric care means those who participated in obstetric care.

^§^Expected number of births = 3.7% (national annual crude birth rate) of the woreda catchment population.

### Proportions of births in all facilities and caesarean sections

Over the course of one year, we recorded 4,231 births at the health institutions. Consequently, 6.6% of the expected 64,413 deliveries occurred at institutions in Gamo Gofa. Table 2 shows the variations in institutional deliveries between the different administrative districts, with the woredas with the largest towns (Arba Minch and Sawla), having the highest proportion of institutional deliveries. When analysing the proportion of institutional deliveries per institutional catchment area, we determined that the proportion varied from zero to an average of > 20% in the two woredas with towns having hospitals. The institutional delivery rate was approximately 3% in areas with health centres not fulfilling the criteria of basic EmOC, while in contrast, areas such as Kamba, with health centres capable of providing EmOC and performing caesarean sections, had a higher rate of institutional deliveries. We used a Pearson product–moment correlation analysis to determine the correlation of the rate of institutional deliveries in the districts to the proportion of midwives in the catchment population of the district and the number of physicians in the districts (where possible). Woredas with a higher ratio of midwives per population (r = 0.71; p < 0.01), and where doctors worked (r =0.66; p < 0.01), were associated with a higher proportion of institutional deliveries.

## Discussion

Based on the total population of 1,740,885, there should have been 14 basic and four comprehensive EmOC facilities in the Zone. There was a sufficient number of health facilities in the Zone, if functional, that could serve as a basic EmOC, and the current study showed that only three basic and two comprehensive EmOC facilities served the population, which is clearly inadequate and below the UN’s minimum recommendations [8]. The proportion of institutional deliveries varied greatly, and five of the health centres did not offer delivery services to the catchment population, whereas 24 health centres provided one or fewer deliveries per month for at least one year. Hence, the population in the area has unequal access to obstetric care. Areas with hospitals and health centres providing comprehensive EmOC had higher rates of institutional deliveries. The number of midwives per population was also an important determining factor for institutional delivery rates.

Another important finding was that 28% of women had abortion complications. Ethiopian law allows an abortion when the pregnancy is due to rape (without the woman being asked to provide evidence of rape), when there is a medical threat to the mother and when the foetus has serious irreversible malformations [12]. The current findings suggest that the women are either unaware of such services, or may have limited access to- and use of contraceptive services.

Our study represents the first mapping of delivery services in a rural Ethiopian district, and the strength of the study was that it included all health institutions in the Gamo Gofa Zone. Although we attempted to record the relevant work being done at the institutions, our data may be incomplete, as facility records of delivery complications and deaths are often incomplete.

Approximately one-fourth of facilities did not provide at least three or four of the signal functions, while 60% did not provide > 5 of the signal functions. This could be because of an inadequate number of trained staff and a lack of the necessary supplies such as medications, blood transfusion bags and resuscitation equipment. Another possible explanation could be the idea that basic emergency obstetric care is rather new, and that these services have not yet been given sufficient priority within the health system. A study conducted in Tanzania has also revealed that there were fewer basic EmOC facilities compared to comprehensive EmOC facilities, which is in contrast to UN standards [13]. A study conducted in a low-income country has shown that many women reported dissatisfaction with unprofessional and careless behaviour at health facilities, and preferred the care of traditional birth attendants or relatives [14].

Our study demonstrated that only 6.6% of expected deliveries occurred at health institutions, while a caesarean section was performed for only 0.8% of the expected births. The overall rate of facility deliveries was even lower without the relatively higher contribution of births in the five better EmOC facilities. The UN minimum is that 10% of expected deliveries should take place in EmOC facilities to help reduce maternal mortality in an area [8]. Additionally, the rates of institutional deliveries varied from one area to the other, thus suggesting unequal use and access to obstetric care. Using the UN guidelines as a reference, both the number of institutional deliveries and caesarean sections were far below what is regarded as adequate in order to reduce maternal deaths [8]. These results agree with earlier research conducted in Ethiopia, as well as in other countries [9,14]. The possible reasons for these findings could be because people live far away from adequately functioning institutions or because of social and cultural restrictions for women to use health institutions during deliveries [15]. Our data suggest that most of the institutions do not provide essential delivery services, and a lack of availability of services near the patients’ homes probably explains the low caesarean section rates.

Seventy-nine maternal deaths occurred at the institutions during the one year surveyed, and the case fatality rate among women with obstetric complications was higher than the minimum standard set by the UN [8]. The causes of deaths are similar to studies elsewhere [16], and if the maternal mortality ratio is 590 per 100,000 live births [3], only one in five of expected maternal deaths are recorded at health institutions.

Approximately one-fourth of all deliveries were complicated. This high proportion of complicated deliveries shows that the population seeks care when complications arise during home deliveries. It is therefore recommended that all women with complicated deliveries should be treated in obstetric emergency care facilities; however, with an expected complication rate of 10%, far too few women with complications received adequate care. A qualitative study with informants of 42 maternal deaths in the Gambia highlighted the challenges mothers face to reach lifesaving health facilities. The major barriers described were as follows: an under-estimation of the severity of the complications, a bad experience with the health-care system, a lack of transportation and prolonged transportation [17]. Moreover, large parts of the population in our study area live in remote mountainous areas, far away from the health institutions. The high proportion of obstetric complications and high maternal death rates in some institutions suggest that health centres do not refer such cases to places where the proper management of complicated births are available. It may also show that there is a lack of trained personnel who can provide correct interventions.

We have noted that remote rural districts without institutions doing comprehensive or basic EmOC have lower institutional deliveries, which is consistent with previous a study in a low-income country [13]. The distance to health services exerts a dual influence on use, as a disincentive to seeking care first and as an obstacle to reaching care after a decision has been made to seek care [14]. Although we showed an inadequate coverage of delivery services, these results can be used as baseline data for planning, improving and implementing delivery services in rural Ethiopia. Studies have shown that the UN guidelines to assess the process indicators have been proven to be generally effective in identifying the level of emergency obstetric care [18]. We suggest that similar studies should be conducted at all zones in the country, and that such information should be used to improve the coverage and quality of health services.

## Conclusion

Our study showed that the availability, use and quality of basic and comprehensive EmOC facilities fell below the accepted standard. This poses a formidable challenge to achieving the MDG related to maternal health. Many women visiting health facilities with complications after abortions need closer attention. Nonetheless, we find it encouraging that current efforts by the public health authorities to use emergency obstetric care guidelines for improving health care in resource-poor settings, and the works to help strengthen the referral system. It is also encouraging to learn a new effort by the Ethiopian government to set up primary hospitals for every 100,000 of the population, thereby improving access to health care.

## Competing interests

The authors declare that they have no competing interests.

## Authors’ contributions

MG conceived the study, coordinated data collection, analysed and interpreted the data, and prepared the draft manuscript. YY conceived the study, helped to organize the data collection, analysed and interpreted the data, and prepared the draft manuscript. EG and YB supervised MG’s master thesis, and took part in the data collection, data analysis, and writing of the paper. BL conceived the study, advised on the data collection, interpreted the data, and helped to write the manuscript. All the authors have read and approved the submitted version of the manuscript.

## Pre-publication history

The pre-publication history for this paper can be accessed here:

http://www.biomedcentral.com/1472-6963/13/459/prepub
